# Manual vacuum aspiration (women's MVA) for endometrial biopsy for patients with suspected endometrial malignancies

**DOI:** 10.1111/jog.15403

**Published:** 2022-08-29

**Authors:** Etsuko Saito, Yoko Matsumoto, Satoshi Nitta, Saho Fujino, Tetsushi Tsuruga, Mayuyo Mori‐Uchino, Haruko Iwase, Takahiro Kasamatsu, Koji Kugu, Yutaka Osuga

**Affiliations:** ^1^ Department of Obstetrics and Gynecology Tokyo Metropolitan Bokutoh Hospital Tokyo Japan; ^2^ Department of Obstetrics and Gynecology, Faculty of Medicine The University of Tokyo Tokyo Japan; ^3^ Department of Obstetrics National Center for Child Health and Development Tokyo Japan

**Keywords:** atypical endometrial hyperplasia, biopsy, endometrial cancer, uterine diseases, vacuum curettage

## Abstract

**Aim:**

Endometrial biopsy is generally performed with a metal uterine curette sonde; however, recently, many types of vacuum aspirators are available, including the manual vacuum aspiration (MVA) system. We used the women's MVA system for endometrial sampling and evaluated its effectiveness in determining the presence of endometrial malignancy.

**Methods:**

Forty‐seven samples were examined using the following procedures after measuring endometrial thickness by transvaginal ultrasonography: fractional curettage biopsy (Bx; 20 samples), total curettage under general anesthesia (T/C; 13 samples), and MVA (14 samples). The quality of the endometrial samples was classified into four types: 1–4, where 1 denoted poor and 4, good quality.

**Results:**

The mean score of the MVA group was significantly higher than that of the partial curettage biopsy group (*p* = 0.0065). No differences were observed between the MVA and total curettage groups (*p* = 1.00). When patients were divided into two groups according to endometrial thickness (<10 mm or ≥10 mm) and analyzed, both the MVA and T/C groups did not show a significant difference in their scores compared to the Bx group when the endometrial thickness was <10 mm. However, when the endometrial thickness was ≥10 mm, the MVA and T/C groups had significantly better scores than the Bx group (*p* = 0.0225 and *p* = 0.0244, respectively). Vagal reflex, as an adverse event, was observed only in two patients in the Bx group (2/20, 10%).

**Conclusion:**

Considering its quality and safety, Karman‐type MVA for endometrial sampling could be an alternative to fractional curettage using a metallic uterine curette sonde.

## Introduction

The incidence of uterine cancer has increased in many countries in recent years, including Japan. In 2018, the number of endometrial cancer cases was 17 089, which is the largest number for a gynecologic malignancy, surpassing 10 978 for cervical cancer and 13 049 for ovarian cancer. The age‐standardized mortality rate of endometrial cancer in 2019 was 1.5 (per 100 000). Endometrial cells and tissues can be collected from patients with abnormal uterine bleeding in outpatient clinics.^1^

Conventionally, endometrial tissue collection is often performed by sharp curettage involving the use of a uterine curette sonde, and the rate of false results due to the location of the disease is high. The use of fractional curettage, which involves repeated tissue collection in four directions, is, thus, recommended.

However, fractional curettage is sometimes very painful and may cause a decrease in blood pressure due to vagal reflex. Furthermore, it is not always easy and safe to carry out the procedure because of the risk of complications, such as uterine perforation due to blind operation with a thin and hard instrument.^2^ Since the 1990s, manual vacuum aspiration (MVA) has become widespread overseas, and in Japan, the MVA system was approved in 2015. WHO's “Safe abortion 2nd edition” recommends the use of MVA as a safe surgical procedure for early pregnancy abortion in terms of reducing complications and protecting the endometrium.^3^ In the near future, suction‐based abortion procedures such as electric vacuum aspiration (EVA) and MVA are expected to become the mainstream procedures for early abortions in Japan.

Endometrial tissue samples obtained by MVA are already being used for diagnostic purposes overseas, and many reports on the diagnostic accuracy and safety have been published.^4^, ^5^ However, there are still very few reports on endometrial biopsy using MVA from Japanese facilities. Therefore, the purpose of our study was to examine the quality of the pathological samples obtained by MVA and the safety of the procedure.

## Materials and Methods

### Patients

Forty‐seven patients whose endometrial tissue was collected from May 2018 to August 2021 at the University of Tokyo Hospital and Tokyo Metropolitan Bokutoh Hospital under informed consent were selected and analyzed with the approval of the ethics committee (No. 3084‐(3) University of Tokyo Hospital; No. 03‐004 Bokutoh Hospital). The clinical diagnoses and suspicions that required endometrial sampling included endometrial cancer and atypical endometrial hyperplasia.

### Samples

Three collection methods were compared: fractional curettage biopsy (Bx; 20 samples), total curettage under general anesthesia (T/C; 13 samples), and MVA (14 samples). Before carrying out the sampling procedures, we measured the endometrial thickness in all the patients by transvaginal ultrasonography.

T/C were performed to those who required them according to the conventional treatment policy. Regarding Bx, with few exceptions, whether to use curette sonde or MVA was determined by the period in which the procedure was performed. The test period was 6 months from May to October 2018 for The University of Tokyo Hospital, and 2 months from July to August 2021 for the Tokyo Metropolitan Bokutoh Hospital.

**FIGURE 1:**
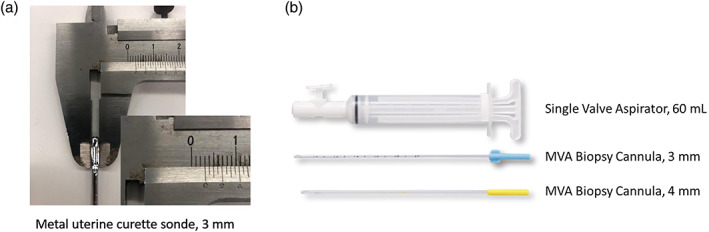
Items included in Karman‐type “women's MVA system” and a metal uterine curette sonde. (a) Thickness of uterine curette sonde was 3 mm. (b) A hard 3‐mm or a soft 4‐mm cannula was combined with a 60‐ml aspirator for the procedure.

Bx was performed in the outpatient setting with a 3 mm sized metal uterine curette sonde (Figure 1a) in four directions of the endometrium. T/C was performed by dilating the cervix under general anesthesia in the operating room, followed by curettage in all directions with a metal curette. As for MVA, we used a Karman‐type “women's MVA system” (Figure 1b), including a disposable kit prepared for uterine endometrial sampling.^6^ The MVA procedure was performed in the outpatient setting without anesthesia, and the samples were collected by a single suction procedure. The MVA was prepared according to the manufacturer's instructions, and the practitioner selected a cannula (3 or 4 mm) for each patient. The 3‐mm cannula is made of slightly harder material than the 4 mm cannula. The cannula was inserted from the uterine ostium to the bottom of the uterus to release the vacuum. The cannula and aspirator were slowly pulled out with spiral rotation to obtain endometrial tissue from all directions. The air inside the aspirator was pushed out into a petri dish containing approximately 10 ml of saline solution, and approximately 10 ml of air was sucked in, and the whole aspirator was shaken lightly to push out the intima tissue adhering to the inner wall of the aspirator with air. Endometrial tissue floating in the saline solution of the Petri dish was transferred to formalin with tweezers. The MVA procedure was simple, and several doctors were able to use the device without problems after observing the expert doctor performing the procedure once.

The metal uterine curette sonde was 3 mm in thickness, which was the same as the harder type of cannula of the MVA. Therefore, there was no case in which we could only collect endometrial tissue with a metal sonde.

**FIGURE 2:**
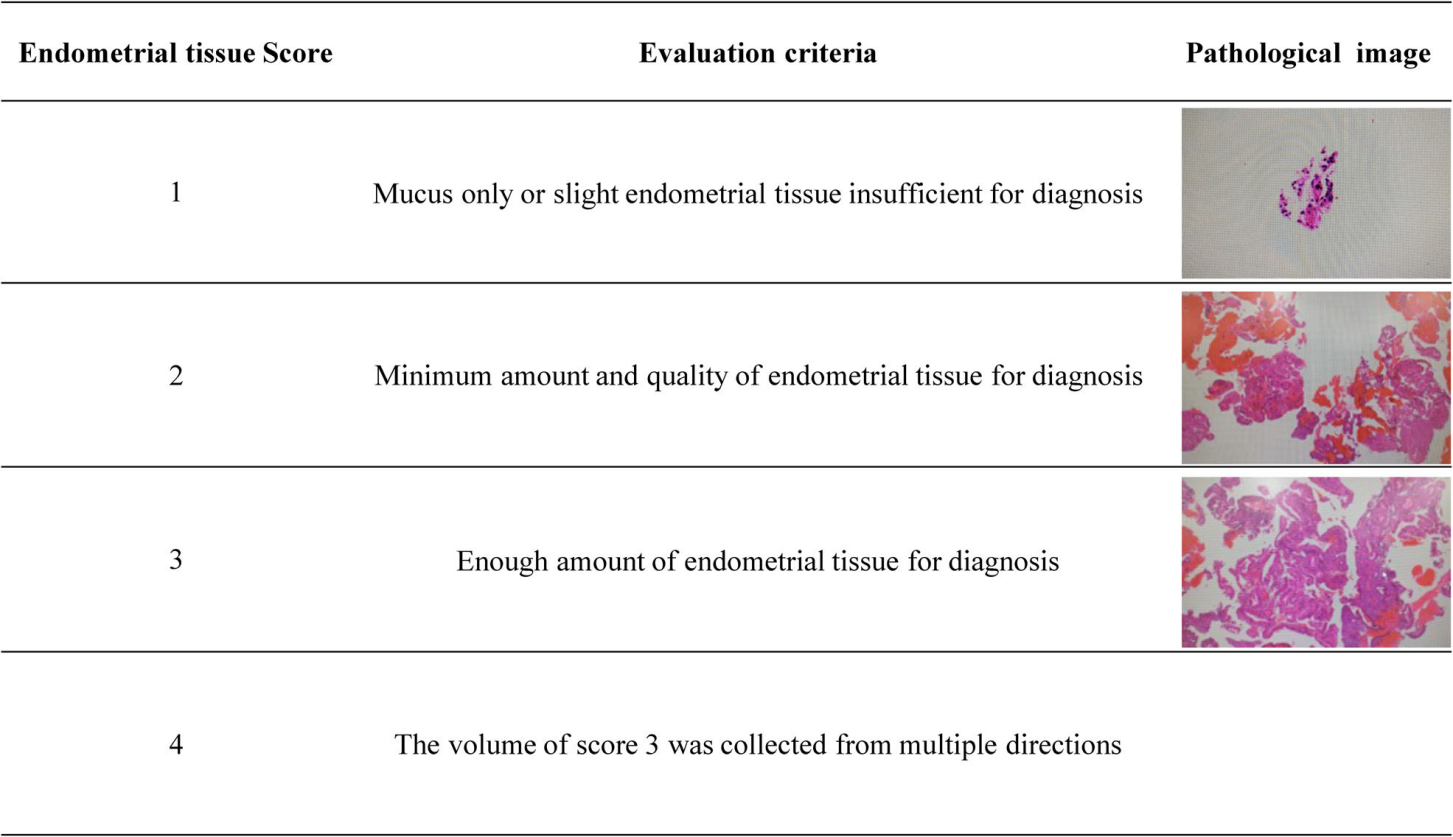
Endometrial tissue score. The amount of endometrial tissue collected by each method was scored from 1 to 4 according to the amount and quality of tissue after hematoxylin and eosin staining for pathological diagnosis.

Tissue analysis was carried out using pathological slides prepared from the collected endometrial tissue, and the amount and quality of the slides was scored according to the criteria shown in Figure 2. A score of 1 indicated that the amount of tissue was too small, and the pathological diagnosis was difficult or unsuccessful, while a score of 4 indicated that the amount was sufficient for successful diagnosis. The scoring of the hematoxylin and eosin (H&E) staining of the samples is also shown in the table. A score of 2 or larger is desirable for pathological diagnosis. Furthermore, 3 and 4 are advantageous for diagnosis. By comparing the average score of tissue collected by MVA with other two procedures, we analyzed the effectiveness of MVA. A researcher, independent of the study, performed the scoring.

### Statistics

The Kruskal–Wallis *H*‐test and Mann–Whitney *U*‐test with Bonferroni correction were used for statistical analyses, and statistically significant differences were considered at *p* values lower than 0.05. Statistical analyses were performed using Excel® Statistical Program File ystat2008.xls for Windows/Macintosh programed by Shinya Yamazaki DDS, PhD, and GraphPad PRISM version 5.0c.

## Results

### Patient background

The background characteristics of the 47 patients are shown in Table 1. Patients already diagnosed with or suspected of endometrial malignancy, including atypical endometrial hyperplasia and endometrial cancer, by diagnostic imaging or cytology were enrolled. The average age was 46.1 ± 12.5 years (range, 28–92 years). The average thickness of the endometrium before the examination was 12.4 ± 8.3 mm (range, 3–35 mm). The patients were divided into two groups based on the endometrial thickness measured using transvaginal ultrasonography: thin endometrium (<10 mm) and thick endometrium (≥10 mm).^7^ There were no significant differences in patient background between the two groups (Table 1). Cases of complete cervical obstruction were excluded from this study because endometrial tissue collection was not possible.

**TABLE 1:** Patient characteristics (*n* = 47)

	Bx	T/C	MVA
*n* (all)	20	13	14
*n* (nulliparous patient)	11	11	6
*n* (post‐menopausal patient)	4	2	8
Age (mean ± *SD*)	28–70 (45.4 ± 11.3)	28–59 (41.7 ± 15.1)	28–92 (51.5 ± 8.8)
Endometrium thickness measurements (mean ± *SD*, mm)	3–34 (12.2)	3–26 (9.2)	3–35 (15.8)
Endometrium thickness <10 mm			
*n*	8	8	5
Age	28–59 (40.1 ± 10.2)	28–59 (38.5 ± 8.7)	28–92 (54.0 ± 23.0)
Endometrium thickness measurements	3–8 (5.9 ± 2.0)	3–9 (5.9 ± 1.8)	3–9 (5.4 ± 2.1)
Endometrium thickness ≥10 mm			
*n*	12	5	9
Age	31–70 (51.5 ± 15.2)	40–57 (46.2 ± 6.4)	36–62 (50.1 ± 7.7)
Endometrium thickness measurements	10–34 (16.4 ± 6.1)	10–26 (14.6 ± 5.9)	11–35 (21.5 ± 8.4)
Diagnosis before examination			
Cancer/cancer suspected	19	12	10
Atypical endometrial hyperplasia/atypical endometrial hyperplasia suspected	1	1	4

*Note*: Age, endometrium thickness, and suspected disease before examination.

Abbreviations: Bx, fractional curettage biopsy; MVA, manual vacuum aspiration; T/C, total curettage.

### Endometrial tissue score analysis

Table 2 shows the average scores for each collection method and the numbers of cases with scores from 1 to 4. The percentages of patients with a score of 3 or 4 were 35% (7/20) and 84.6% (11/13) and 85.7% (12/20) in the Bx group and T/C and MVA groups, respectively. No sample had a score of 1 in the curettage group, but one sample each in the Bx and MVA groups did—the endometrial thickness of those two patients was 3 mm.

**TABLE 2:** Comparison of endometrial tissue volume score by procedure and pathological results

	Bx	T/C	MVA	*p* value^a^
All patient score	1–3 (2.3 ± 0.6)	2–4 (3.5 ± 0.8)	1–4 (3.4 ± 0.9)	0.0001
Score 1 cases	1 (5%)	0 (%)	1 (7.1%)	
Score 2 cases	12 (50%)	2 (15.4%)	1 (7.1%)	
Score 3 cases	7 (35%)	2 (15.4%)	4 (28.6%)	
Score 4 cases	0 (0%)	9 (69.2%)	8 (57.1%)	
Endometrium thickness (<10 mm)	1–3 (2.3 ± 0.7)	2–4 (3.4 ± 0.9)	1–4 (3.2 ± 1.3)	0.0364
Score 1 cases	1 (12.5%)	0 (0%)	1 (20%)	
Score 2 cases	4 (50%)	2 (25%)	0 (0%)	
Score 3 cases	3 (37.5%)	1 (12.5%)	1 (20%)	
Score 4 cases	0 (0%)	5 (62.5%)	3 (60%)	
Endometrium thickness (≥10 mm)	1–3 (2.3 ± 0.5)	3–4 (3.8 ± 0.4)	2–4 (3.4 ± 0.7)	0.0008
Score 1 cases	0 (0%)	0 (0%)	0 (0%)	
Score 2 cases	8 (66.7%)	0 (0%)	1 (11.1%)	
Score 3 cases	4 (33.3%)	1 (20%)	3 (33.3%)	
Score 4 cases	0 (0%)	4 (80%)	5 (55.5%)	
Diagnosis after the examination				
Normal findings or no malignant findings	7	2	5	
Atypical endometrial hyperplasia	9	3	2	
Cancer	4	8	6	
Undecidable	0	0	1	

^a^ Comparison of three groups using Kruskal–Wallis *H*‐test.

**FIGURE 3:**
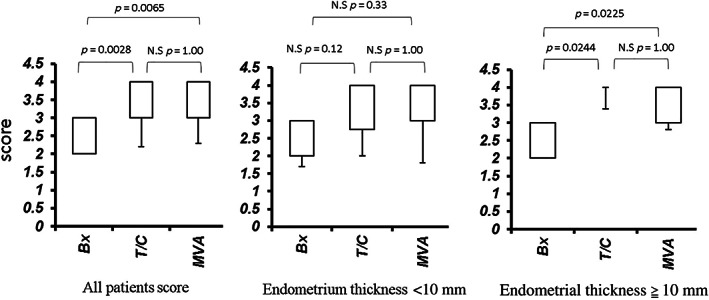
Comparison of endometrial tissue volume score by procedure. Two‐group comparison: Mann–Whitney *U*‐test with Bonferroni correction.

When the scores of the three collection methods were compared without considering endometrial thickness, the MVA group had significantly higher scores than the biopsy group (*p* = 0.0065; Figure 3). No difference was observed between the MVA and T/C group (*p* = 1.00). When the data were analyzed according to endometrial thickness of 10 mm or more and less than 10 mm, there was a significant difference in the scores between the Bx and MVA or T/C of thick endometrial group (*p* = 0.0225 and *p* = 0.0244, respectively). However, in the thin endometrial group, no significant differences were observed between Bx and MVA or T/C (*p* = 0.33 and *p* = 0.12, respectively) (Figure 3).

Pathological diagnosis was difficult in one case in the MVA group because of low tissue volume. Since the endometrium was 3 mm thick, and only necrotic tissue was collected, a pathological diagnosis could not be made. Other than that, diagnoses were made in all groups. There was also a sample with a score of 1 in the Bx group, but the pathologist managed to make a diagnosis of no malignancy with that sample.

In the Bx group, adverse events were observed in two patients (2/20, 10%). Decreased blood pressure and nausea due to vagal reflex were observed in one patient, and decreased blood pressure was observed in one patient. The patients recovered after a short rest with no additional medication. The two patients with side effects were both nulliparous and premenopausal patients. In contrast, no adverse events were observed in the MVA group. No uterine perforation was observed during any of the procedures.

## Discussion

We found that the quality of endometrial tissue collected by MVA was comparable to that collected by T/C. The effectiveness of MVA has been reported in many papers. Tansathit et al. examined the diagnostic efficacy of endometrial aspiration histology (Karman‐type cannula = women's MVA) with a study sample of 226 patients with abnormal bleeding.^8^ The sensitivity of MVA was 86.7% and specificity was 100% compared to curettage sampling.

In the study by Sirimai et al., endometrium samples were collected using both conventional metal curettage and MVA from 132 patients with abnormal bleeding, and the pathological results were compared. The diagnostic concordance rate among the 123 evaluable samples was 64.2%. Atypical proliferation (one case) and cancer (five cases) could be diagnosed consistently using either method.^9^

Regarding the cannula size, the size of 3 mm was selected for patients whose uterine ostium and cervical canal were narrow. For patients with a wide uterine ostium/lumen and with a complicated direction of the endometrium, the size of 4 mm was selected. Thus, smooth tissue collection was possible. In addition, while the safety of MVA was not inferior to that of conventional fractional curettage, this needs to be investigated in future studies with larger sample sizes. Once accustomed to the procedure, both the practitioner and the caregiver took almost the same amount of time and effort for MVA as they did for fractional curettage.

Notable complications of endometrial fractional curettage include uterine perforation and vagal reflex. The risk of uterine perforation due to fractional curettage or dilatation and curettage (D&C) is estimated to be up to 1%, with an incidence of 0.16%–0.9%,^10^, ^11^ although the precise rates for each procedure are not clear. Vagal reflex is also a common complication of uterine examination, while the precise risks for each procedure are unknown, there is an estimated risk of up to 2% when using hard equipment such as a rigid hysteroscope compared with a flexible hysteroscope.^12^, ^13^ These reports suggest that the use of MVA for endometrial biopsy in outpatient clinics could result in a decrease in vasovagal reflex. In our study, vagal reflex was observed in 10% of patients in the Bx group but in 0% of the patients in the MVA group. While the difference was not significant because the number of patients was small, MVA may be safer than conventional methods in terms of vagal reflex.

The limitation of this study would be the small number of samples, which might result in no significant difference in analyses of thin endometrial patients. Further study with more samples would be needed to evaluate the precise advantage, adverse events, and pathology quality, for example, by following patients' conditions after leaving the hospital. Furthermore, it would also be necessary to consider the impact of MVA on cost‐effectiveness.

Some studies have reported that D&C is more accurate than aspiration biopsy for patients with endometrial hyperplasia^14^ and for the follow‐up evaluation of patients treated with progestin for endometrial hyperplasia.^15^ However, we found that MVA, which can be performed in the outpatient setting, was a beneficial alternative to fractional curettage using a metallic uterine curette sonde. Furthermore, MVA may also be an alternative to the T/C procedure, which is performed under anesthesia, although further studies with careful selection of the indications and patients are needed. Finally, when it is necessary to collect the endometrium on a regular basis, for instance, to determine the effect of high‐dose medroxyprogesterone acetate therapy for fertility preservation, the burden on the patient may be reduced with the use of MVA.

## Conflict of Interest

The authors have no conflict of interest to declare.

## Data Availability

The data that support the findings of this study are available from the corresponding author upon reasonable request.
